# Supplementary material for: Pyruvate Dehydrogenase Kinase 1 inhibition mediated oxidative phosphorylation enhancement in cartilage promotes osteoarthritis progression
Source: BMC Musculoskelet Disord. 2023 Jul 20;24:597. doi: 10.1186/s12891-023-06585-6 (PMC10357736; doi:10.1186/s12891-023-06585-6)
Supplement: Supplementary file 1 — Additional file 1. [file 12891_2023_6585_MOESM1_ESM.zip › MF.Gsea.1653627316242/GOMF_SCAFFOLD_PROTEIN_BINDING.html]

Details for gene set GOMF\_SCAFFOLD\_PROTEIN\_BINDING[GSEA]

|  || Dataset | 55457\_collapsed\_to\_symbols.cls.cls #OA\_versus\_normal.cls.cls #OA\_versus\_normal\_repos |
| Phenotype | cls.cls#OA\_versus\_normal\_repos |
| Upregulated in class | normal |
| GeneSet | GOMF\_SCAFFOLD\_PROTEIN\_BINDING |
| Enrichment Score (ES) | -0.35686126 |
| Normalized Enrichment Score (NES) | -1.403539 |
| Nominal p-value | 0.047694754 |
| FDR q-value | 0.26634744 |
| FWER p-Value | 1.0 |
Table: GSEA Results Summary

  

Fig 1: Enrichment plot: GOMF\_SCAFFOLD\_PROTEIN\_BINDING      
 Profile of the Running ES Score & Positions of GeneSet Members on the Rank Ordered List

  

| SYMBOL | TITLE | RANK IN GENE LIST | RANK METRIC SCORE | RUNNING ES | CORE ENRICHMENT || 1 | TREM2 | triggering receptor expressed on myeloid cells 2 [Source:HGNC Symbol;Acc:HGNC:17761] | 342 | 0.726 | 0.0100 | No |
| 2 | KCNQ1 | potassium voltage-gated channel subfamily Q member 1 [Source:HGNC Symbol;Acc:HGNC:6294] | 391 | 0.709 | 0.0421 | No |
| 3 | PLCG2 | phospholipase C gamma 2 [Source:HGNC Symbol;Acc:HGNC:9066] | 725 | 0.588 | 0.0459 | No |
| 4 | PARK7 | Parkinsonism associated deglycase [Source:HGNC Symbol;Acc:HGNC:16369] | 1406 | 0.442 | 0.0152 | No |
| 5 | MAP3K7 | mitogen-activated protein kinase kinase kinase 7 [Source:HGNC Symbol;Acc:HGNC:6859] | 1701 | 0.399 | 0.0125 | No |
| 6 | CRK | "CRK proto-oncogene, adaptor protein [Source:HGNC Symbol;Acc:HGNC:2362]" | 2018 | 0.360 | 0.0060 | No |
| 7 | KCNA5 | potassium voltage-gated channel subfamily A member 5 [Source:HGNC Symbol;Acc:HGNC:6224] | 2387 | 0.316 | -0.0067 | No |
| 8 | TREM1 | triggering receptor expressed on myeloid cells 1 [Source:HGNC Symbol;Acc:HGNC:17760] | 2797 | 0.270 | -0.0249 | No |
| 9 | CRIPT | CXXC repeat containing interactor of PDZ3 domain [Source:HGNC Symbol;Acc:HGNC:14312] | 3242 | 0.227 | -0.0481 | No |
| 10 | CDC37 | "cell division cycle 37, HSP90 cochaperone [Source:HGNC Symbol;Acc:HGNC:1735]" | 3810 | 0.175 | -0.0834 | No |
| 11 | NLGN4X | neuroligin 4 X-linked [Source:HGNC Symbol;Acc:HGNC:14287] | 3974 | 0.158 | -0.0881 | No |
| 12 | ATP2B4 | ATPase plasma membrane Ca2+ transporting 4 [Source:HGNC Symbol;Acc:HGNC:817] | 3998 | 0.156 | -0.0820 | No |
| 13 | SYK | spleen associated tyrosine kinase [Source:HGNC Symbol;Acc:HGNC:11491] | 4084 | 0.147 | -0.0812 | No |
| 14 | ITPR2 | "inositol 1,4,5-trisphosphate receptor type 2 [Source:HGNC Symbol;Acc:HGNC:6181]" | 4222 | 0.135 | -0.0850 | No |
| 15 | NOS1 | nitric oxide synthase 1 [Source:HGNC Symbol;Acc:HGNC:7872] | 4228 | 0.134 | -0.0786 | No |
| 16 | CACNA1G | calcium voltage-gated channel subunit alpha1 G [Source:HGNC Symbol;Acc:HGNC:1394] | 4422 | 0.119 | -0.0876 | No |
| 17 | SHANK1 | SH3 and multiple ankyrin repeat domains 1 [Source:HGNC Symbol;Acc:HGNC:15474] | 4511 | 0.112 | -0.0888 | No |
| 18 | XRCC6 | X-ray repair cross complementing 6 [Source:HGNC Symbol;Acc:HGNC:4055] | 4699 | 0.096 | -0.0986 | No |
| 19 | CAV2 | caveolin 2 [Source:HGNC Symbol;Acc:HGNC:1528] | 4924 | 0.078 | -0.1121 | No |
| 20 | IKBKB | inhibitor of nuclear factor kappa B kinase subunit beta [Source:HGNC Symbol;Acc:HGNC:5960] | 5914 | 0.001 | -0.1890 | No |
| 21 | NLGN3 | neuroligin 3 [Source:HGNC Symbol;Acc:HGNC:14289] | 6371 | -0.033 | -0.2229 | No |
| 22 | LYN | "LYN proto-oncogene, Src family tyrosine kinase [Source:HGNC Symbol;Acc:HGNC:6735]" | 6514 | -0.043 | -0.2318 | No |
| 23 | KRT15 | keratin 15 [Source:HGNC Symbol;Acc:HGNC:6421] | 6643 | -0.053 | -0.2391 | No |
| 24 | DSP | desmoplakin [Source:HGNC Symbol;Acc:HGNC:3052] | 6931 | -0.074 | -0.2577 | No |
| 25 | SRC | "SRC proto-oncogene, non-receptor tyrosine kinase [Source:HGNC Symbol;Acc:HGNC:11283]" | 6940 | -0.075 | -0.2545 | No |
| 26 | AKAP5 | A-kinase anchoring protein 5 [Source:HGNC Symbol;Acc:HGNC:375] | 6964 | -0.077 | -0.2524 | No |
| 27 | KRT18 | keratin 18 [Source:HGNC Symbol;Acc:HGNC:6430] | 7027 | -0.082 | -0.2531 | No |
| 28 | PANX1 | pannexin 1 [Source:HGNC Symbol;Acc:HGNC:8599] | 7344 | -0.105 | -0.2724 | No |
| 29 | SCN5A | sodium voltage-gated channel alpha subunit 5 [Source:HGNC Symbol;Acc:HGNC:10593] | 7485 | -0.117 | -0.2774 | No |
| 30 | NLGN1 | neuroligin 1 [Source:HGNC Symbol;Acc:HGNC:14291] | 7602 | -0.127 | -0.2800 | No |
| 31 | CACNA1H | calcium voltage-gated channel subunit alpha1 H [Source:HGNC Symbol;Acc:HGNC:1395] | 7635 | -0.129 | -0.2759 | No |
| 32 | KCNH2 | potassium voltage-gated channel subfamily H member 2 [Source:HGNC Symbol;Acc:HGNC:6251] | 7724 | -0.136 | -0.2759 | No |
| 33 | BRAF | "B-Raf proto-oncogene, serine/threonine kinase [Source:HGNC Symbol;Acc:HGNC:1097]" | 8171 | -0.169 | -0.3022 | No |
| 34 | GRM2 | glutamate metabotropic receptor 2 [Source:HGNC Symbol;Acc:HGNC:4594] | 8425 | -0.187 | -0.3124 | No |
| 35 | LRP4 | LDL receptor related protein 4 [Source:HGNC Symbol;Acc:HGNC:6696] | 8564 | -0.199 | -0.3131 | No |
| 36 | PDE4D | phosphodiesterase 4D [Source:HGNC Symbol;Acc:HGNC:8783] | 8797 | -0.216 | -0.3203 | No |
| 37 | GRID2 | glutamate ionotropic receptor delta type subunit 2 [Source:HGNC Symbol;Acc:HGNC:4576] | 8974 | -0.230 | -0.3224 | No |
| 38 | GJA1 | gap junction protein alpha 1 [Source:HGNC Symbol;Acc:HGNC:4274] | 9325 | -0.258 | -0.3366 | No |
| 39 | CIT | citron rho-interacting serine/threonine kinase [Source:HGNC Symbol;Acc:HGNC:1985] | 9356 | -0.260 | -0.3259 | No |
| 40 | NCK2 | NCK adaptor protein 2 [Source:HGNC Symbol;Acc:HGNC:7665] | 9755 | -0.297 | -0.3419 | Yes |
| 41 | TCOF1 | treacle ribosome biogenesis factor 1 [Source:HGNC Symbol;Acc:HGNC:11654] | 9771 | -0.298 | -0.3280 | Yes |
| 42 | HOMER1 | homer scaffold protein 1 [Source:HGNC Symbol;Acc:HGNC:17512] | 9784 | -0.300 | -0.3138 | Yes |
| 43 | BAIAP2 | BAR/IMD domain containing adaptor protein 2 [Source:HGNC Symbol;Acc:HGNC:947] | 9892 | -0.311 | -0.3064 | Yes |
| 44 | P2RY1 | purinergic receptor P2Y1 [Source:HGNC Symbol;Acc:HGNC:8539] | 9925 | -0.314 | -0.2930 | Yes |
| 45 | MAP2K2 | mitogen-activated protein kinase kinase 2 [Source:HGNC Symbol;Acc:HGNC:6842] | 9932 | -0.315 | -0.2776 | Yes |
| 46 | NOS3 | nitric oxide synthase 3 [Source:HGNC Symbol;Acc:HGNC:7876] | 9980 | -0.319 | -0.2652 | Yes |
| 47 | FYN | "FYN proto-oncogene, Src family tyrosine kinase [Source:HGNC Symbol;Acc:HGNC:4037]" | 10092 | -0.330 | -0.2571 | Yes |
| 48 | MAP2K1 | mitogen-activated protein kinase kinase 1 [Source:HGNC Symbol;Acc:HGNC:6840] | 10197 | -0.340 | -0.2481 | Yes |
| 49 | ATG4B | autophagy related 4B cysteine peptidase [Source:HGNC Symbol;Acc:HGNC:20790] | 10243 | -0.346 | -0.2341 | Yes |
| 50 | CASP8 | caspase 8 [Source:HGNC Symbol;Acc:HGNC:1509] | 10442 | -0.366 | -0.2311 | Yes |
| 51 | CD163 | CD163 molecule [Source:HGNC Symbol;Acc:HGNC:1631] | 11075 | -0.441 | -0.2580 | Yes |
| 52 | DLG4 | discs large MAGUK scaffold protein 4 [Source:HGNC Symbol;Acc:HGNC:2903] | 11077 | -0.441 | -0.2358 | Yes |
| 53 | GRM3 | glutamate metabotropic receptor 3 [Source:HGNC Symbol;Acc:HGNC:4595] | 11351 | -0.478 | -0.2329 | Yes |
| 54 | KRT5 | keratin 5 [Source:HGNC Symbol;Acc:HGNC:6442] | 11588 | -0.513 | -0.2254 | Yes |
| 55 | CHUK | component of inhibitor of nuclear factor kappa B kinase complex [Source:HGNC Symbol;Acc:HGNC:1974] | 11624 | -0.521 | -0.2018 | Yes |
| 56 | KRT8 | keratin 8 [Source:HGNC Symbol;Acc:HGNC:6446] | 11774 | -0.545 | -0.1859 | Yes |
| 57 | YWHAE | tyrosine 3-monooxygenase/tryptophan 5-monooxygenase activation protein epsilon [Source:HGNC Symbol;Acc:HGNC:12851] | 12137 | -0.628 | -0.1824 | Yes |
| 58 | UBTF | upstream binding transcription factor [Source:HGNC Symbol;Acc:HGNC:12511] | 12261 | -0.665 | -0.1584 | Yes |
| 59 | IL6ST | interleukin 6 cytokine family signal transducer [Source:HGNC Symbol;Acc:HGNC:6021] | 12505 | -0.749 | -0.1395 | Yes |
| 60 | NLGN4Y | neuroligin 4 Y-linked [Source:HGNC Symbol;Acc:HGNC:15529] | 12521 | -0.759 | -0.1023 | Yes |
| 61 | PSAP | prosaposin [Source:HGNC Symbol;Acc:HGNC:9498] | 12569 | -0.780 | -0.0666 | Yes |
| 62 | HSP90AA1 | heat shock protein 90 alpha family class A member 1 [Source:HGNC Symbol;Acc:HGNC:5253] | 12730 | -0.893 | -0.0339 | Yes |
| 63 | VIM | vimentin [Source:HGNC Symbol;Acc:HGNC:12692] | 12777 | -0.932 | 0.0096 | Yes |
Table: GSEA details [plain text format]

  

Fig 2: GOMF\_SCAFFOLD\_PROTEIN\_BINDING      
 Blue-Pink O' Gram in the Space of the Analyzed GeneSet

  

Fig 3: GOMF\_SCAFFOLD\_PROTEIN\_BINDING: Random ES distribution      
 Gene set null distribution of ES for **GOMF\_SCAFFOLD\_PROTEIN\_BINDING**
